# The Role of Assistive Technology in Enabling Older Adults to Achieve Independent Living: Past and Future

**DOI:** 10.2196/58846

**Published:** 2024-07-30

**Authors:** Anna Sweeting, Katie A Warncken, Martyn Patel

**Affiliations:** 1 Norwich Institute for Healthy Ageing Norwich Medical School University of East Anglia Norwich United Kingdom; 2 Older Peoples Medicine Department Norfolk and Norwich University Hospital NHS Foundation Trust Norwich United Kingdom

**Keywords:** assistive technology, older adults, users, aging, aging in place, UK, cocreation, research trial, independent living, North Norfolk, disability, injury, tool, use, design, barrier

## Abstract

In this viewpoint, we present evidence of a marked increase in the use of assistive technology (AT) by older adults over the last 25 years. We also explain the way in which this use has expanded not only as an increase in terms of the total number of users but also by going beyond the typical scopes of use from its inception in 1999 to reach new categories of users. We outline our opinions on some of the key driving forces behind this expansion, such as population demographic changes, technological advances, and the promotion of AT as a means to enable older adults to achieve independent living. As well as our review of the evolution of AT over the past 25 years, we also discuss the future of AT research as a field and the need for harmonization of terminology in AT research. Finally, we outline how our experience in North Norfolk (notably the United Kingdom’s most old age–dependent district) suggests that cocreation may be the key to not only successful research trials in the field of AT but also to the successful sustained adoption of AT beyond its original scope of use.

## Spectacular Increase in Assistive Technology Use and Published Research Over the Last 25 Years

Practitioners in the field of assistive technology (AT) will be well versed in how over the last quarter of a century there has been a marked increase in the use of traditional or historically available AT (referring to the type of AT available prior to 2000) aimed at assisting those with a disability or specific injury, while acknowledging that some important barriers to access remain [1,2]. Here, we discuss how equity disparities in the access to AT vary globally and how countries facing disproportionately higher growth among the demographic of older adults may experience the least opportunity of access.

The same practitioners will also be aware that the number and type of different technologies being applied in the field of AT developed since the turn of the millennium have increased at an even greater pace than the rate of increase in the total amount of AT used [3]. In this viewpoint, we will cover the plethora of categories of AT available and highlight how comparing trials in the field of AT is a challenge due to heterogeneity in related terminology and research outcomes.

Notably, over this 25-year span of time, what has increased most of all is the size of the potential user pool, defined as potential users who require AT to enhance their lives. This user pool includes adults of all ages living with specific disease-related disability or those specifically seeking support for independent living, including older adults with nondisease-specific frailty for whom AT is a powerful tool to facilitate aging in place [4,5]. Throughout this article we will use local, regional, and global research evidence to draw a line between the increase in potential users and growth of AT as a research field and the potential for AT to enable older adults to achieve independent living. We will also discuss how, in our opinion, cocreation may be the most valuable tool in enhancing the uptake of AT.

## Growth of AT Research

**Figure 1:**
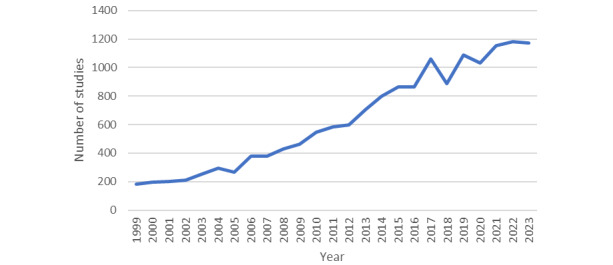
Number of research articles found per year between 1999 and 2023 by searching for "assistive technology" on PubMed.

Over the last 25 years, AT as a research field has witnessed a nearly 10-fold increase [6]. Using search returns for “assistive technology” on PubMed as a crude measure of research interest, in 1999, there were 181 reported studies; by 2009, that number had risen to 463 studies; and by 2019, the number of studies more than doubled again to 1089 (Figure 1) [7]. Clearly, these data alone can only speak to a trend in the increase in research output within the field of AT and not to the quality or impact of this research. There remain many unmet questions in AT research, with a recent review of the state of European AT provision noting the lack of both comprehensive data on AT provision and coverage and consistency in terminology, making direct comparisons a challenge [8].

## Defining AT

There are several definitions of AT, with some variance internationally. The UK definition used by the National Institute for Health and Care Research is as follows [9]:

Products or systems that support and help individuals with disabilities, restricted mobility or other impairments to perform functions that might otherwise be difficult or impossible. These devices support individuals to improve or maintain their daily quality of life by easing or compensating for an injury or disability.

Within this definition, “other impairments” to performing activities of daily living can include frailty associated with old age, while many other definitions have now encompassed general frailty as well as condition-specific disabilities [10].

Having defined AT, we will now compare and contrast the state of the AT field in 1999 and in 2024.

## State of AT in 1999

Typically, AT that was available around 1999—and this is by no means an exhaustive list—includes categories such as adapted home items (eg, foam padded cutlery), mobility devices (eg, walking frames and wheelchairs), sensory support (eg, hearing aid devices), telemonitoring systems (eg, pendant alarm services that connect to analog phone lines), and enhanced communication devices (eg, text to speech) [3].

Browsing the 181 studies published in 1999 retrieved from PubMed gives us some insight into where the focus of most of the research output in the field was at that point. Of those studies, 93 focused in some way on mobility using wheelchairs for those with specific disabilities, including reviews of advances in the field [11], and 35 focused on communication enhancement using assistive devices, such as those used in a school environment [12].

## Twenty-Five Years Later: State of AT Today

An updated list of AT available in 2024 that was not available in 1999 (again, this is a representative and not an exhaustive list) would include [13-17] telemonitoring systems (eg, fall detector alarm services that connect via Wi-Fi); the Internet of Things, defined as any device that has added functionality due to an ability to connect to the internet; ambient/active assisted living robots, which involves the use of passive monitoring devices that can also be combined with feedback effector mechanisms; GPS-based monitoring via a bespoke device or via an app on a smartphone; smart home technology, which involves the use of passive sensor technology (eg, infrared sensors for motion detection) to keep track of surrogate markers of function in a defined environment; and smartphone apps to promote maintenance of function (eg, apps for visual impairment or physical activity trackers).

It is harder to pin down a single majority topic for the 1089 AT-related studies published in 2019, but it is clear that there is a much greater emphasis on the use and innovation afforded by new technologies, along with more studies that address the use of AT for older adults without a specific disability, asking questions such as “can robots replace carers?” [18]. Estimates for the number of users of AT are hard to obtain, given that AT is provided by many different means (eg, health care contact, social care contact, and the open market). However, as an illustration, in Norfolk, United Kingdom, there are an estimated 12,000 users of AT provided by the adult social care sector, representing roughly 2% of the total adult population (personal communication, C Metcalf, Assistive Technology County Manager, Adult Social Care at Norfolk County Council; email correspondence May 26, 2023).

Some commonalities of AT do exist. The AT of 2019 was more likely to connect to other devices or the internet, to contain a microprocessor chip, and to require the user to have access to a broadband communications network. If we were to narrow the focus to developments in the last year, the AT-related articles published in 2024 start to increasingly include references to artificial intelligence (AI) in their scope, representing a global “arms race” to find ways to use this nascent technology [14].

However, it would be a false dichotomy to state that the pools of available AT in 1999 and 2024 are entirely separate from each other. There is of course a continuum between the two phases. Some AT tools that are currently on the market are more or less exactly the same as those available in 1999 (eg, personal falls alarms), whereas some have incrementally changed but would still be recognizable as the same product (eg, wheelchairs). However, there is some AT available today that bears no relation to the AT of 1999. We will give examples of these technologies below when discussing the current categories of AT, but first we must discuss how the experience of AT access may vary globally in response to changes in population demographics.

## Actual and Projected Increase of Older Adults (Especially in Lower- and Middle-Income Countries)

One may hypothesize that the single largest driver in the expansion of AT is the global change in population demographics, with the number of older adults increasing (and projected to continue to increase) in both total numbers and as a percentage of overall populations worldwide, as demonstrated in a World Health Organization report [19]. There is evidence to support this claim within high-income countries, where measures of increased dependency are matched by increased AT use [20]. As has been pointed out, this growth is not equal in all countries, and there is a disappointing disparity in that lower- and middle-income countries that have the fastest expansion rates of older adults are more likely to have the least ready access to AT or a substantial body of AT research [21]. Furthermore, in 2019, an analysis of disease burden data showed that the statistical evidence at the global population level demonstrates a disappointing maintenance of average disability levels in the population 70 years and older to date [22].

Given the large and expanding number of individuals aged over 70 years with disability worldwide, there have been calls for designers of new AT, especially in the field of AI, to pay particular attention to factors affecting equity of access and thus enable AT to realize its full potential in the global market [23].

To substantiate this hypothesis, we have to also answer the following question: Why does future care have to be technologically based rather than having more human carers?

## The Flip Side of an Aging Population: Not Enough Carers, or the Old Age Dependency Ratio

Alongside an increase in older adults comes an urgent need for solutions to care problems that are not human-focused. As the number of adults in retirement increases, the corollary is that the percentage of adults of working age available (or willing) to work in caring roles decreases [19,24]. Put simply, not only are there currently not enough carers, but as birth rates decline in many nations, barring migration, there is not likely to be a positive change in workforce availability at an individual country level [25]. Therefore, the demand for AT to fill a gap as a nonhuman solution to (some) traditional care needs has expanded the categories of use that AT might occupy [13,14].

This lack of carer availability creates a bottleneck pressure, as evidenced within the British health care system, with hospital bed days occupied by patients waiting for care increasing year on year, accounting for 1 in 7 occupied beds at points of peak pressure [26].

In the United Kingdom, North Norfolk is the district (geographical unit below the county size) with the highest old age dependency ratio (OADR), which refers to the number of people of State Pension age (SPA; currently set at 60 years for women and 65 years for men) and older for every 1000 people aged between 16 years and up to the SPA [27]. For North Norfolk (a coastal district with no large urban centers), the OADR is currently at 607, whereas South Cambridgeshire (a district with a large student population) has an OADR of 316 and Southwark (a central London district) has an OADR of only 113. The highest OADR clusters occur in rural and coastal communities, and this ratio is highlighted as a particular driver of health inequality [28].

## Inconsistent AT Definitions: Risks of Too Many Categories

Before turning our attention to some of the structural drivers of the expansion of AT, it is important to spend a moment digressing on the harmonization of terminology. It is our opinion that a field that does not agree on set definitions of terms and research outcomes risks fracturing. A fractured field loses the ability to present a unified front to policy makers.

There is no set agreed-upon method for subcategorizing AT; as has been noted, this multiplicity of descriptive categorizations in the research literature risks holding back the field due to the potential for duplication of work with overlapping categories and an inherent challenge in comparing 2 or more studies that use very different categorization techniques [3,5]. Similar challenges have been tackled in other emergent research fields in the last quarter-century, particularly in frailty research, where organizations such as the Global Leadership Initiative on Sarcopenia have sought to harmonize definitions [29]. Perhaps a similar exercise in setting international standards for AT research is required.

Interestingly, this challenge of developing a shared terminology may be an inherent property reflecting the way in which AT has expanded, in that we see not only more and better versions of historic AT products but also an increase in the variety, complexity, and interactivity of AT products [13-17,21]. The AT development pathway has adopted general technological developments since the turn of the millennium, including GPS technology, information connectivity, and miniaturization of computer chips to name only a few relevant fields.

The complexity of categorization of AT may be influenced by the arena of discussion. When introducing the concept of AT subcategories in patient and public focus groups, we prefer simplicity, such as this 4-category definition outlined in a recent systematic review [5]: (1) AT that enables accessible communication (eg, closed-caption technology [CCT]), (2) AT that triggers a request for emergency assistance (eg, falls alarm technology), (3) AT that promotes or improves physical well-being (eg, step counters or pedometers), and (4) AT that promotes or improves mental well-being (eg, mindfulness apps).

This categorization enables the quick introduction of a new concept over a short period of time by using a broader definition set. Equally, when discussing the nuances of the myriad technologies deployed in AT, a research methods paper may have 10 or more categories [3].

Having discussed the choices in different formats of categorization, we now return to our main argument of outlining the reasons driving the changes in AT over the last 25 years. We have presented above how the use of AT has increased over time and how the population most likely to use AT has increased and will increase further; below, we lay out what are, in our opinion, the 3 key drivers of the evolution of AT available.

## Three Factors Driving the Need for Changes in AT Over Time

### Phasing Out of Analog and Phasing In of Digital Technology

One particular challenge/opportunity of AT development has been heralded by the analog copper wire phone line technology switch-off schedule in the United Kingdom, which is due to be completed by 2025 [30]. The UK government has challenged the AT market to derive a benefit to service users from the switch from analog to digital phone lines, which is a significant challenge to industry given the large section of AT products (such as personal fall alarm sensors) that are reliant on phone lines to function. As most households in the United Kingdom now have either wired-in broadband or 4G network Wi-Fi via smartphones (ie, wireless internet connectivity), we have clearly reached a tipping point where the reliance on copper wire technology no longer makes sense [31].

### Broadening of Scope

We have covered the change in global population demographics that has led to a greater proportion of older adults; however, we also need to recognize that the types of older adults that may benefit from AT have also expanded due to a deliberate broadening of the scope of use of AT. While much of the available AT will always be used to support older adults with a condition- or injury-specific disability, the purpose of AT use has expanded to include that of assisting older adults to maintain an independent living environment in their own homes, also known as aging in place, with many reviews dedicated to this topic [6].

This concept of aging in place has gained substantial traction with policy makers to the extent that it has entered the official lexicon of government, with a typical definition as follows [32]: “The ability to live in one’s own home and community safely, independently, and comfortably, regardless of age, income, or ability level.”

Therefore, we can see that AT advances are not only driven by the changes in the technological landscape but also by the broadening of scope of use of AT to help solve what is arguably the biggest societal challenge of our day [4]. Crucially, this broadening of scope is more than simply the inevitable product of combining the trends of more older adults along with declining carer availability, but can also be seen as a positive expression of a societally determined response to the success of midlife health care. This in turn creates the opportunity to extend the healthy and independent fraction of the lifespan rather than creating a prolonged preterminal/dependent “last act” to our lives [5,6].

### Cross-Fertilization of Ideas With Mainstream Society

Another driver of the profile of AT has been the adoption of some ideas that started in AT for widespread use in mainstream society [3]. A key example of this cross-over in use is provided by CCT, which started initially as a service for the deaf community. CCT use has now spread further than its creators could have envisaged, as it is commonly used by many adults without a hearing impairment when wishing to stream video content silently (eg, in a shared-use or public space) or to pick up a new language quickly. Furthermore, there is evidence of a developmental benefit of CCT, in that children who watch videos with both audio and CCT switched on achieve reading milestones earlier than those using only audio [33]. This exchange of ideas is bidirectional, with mainstream GPS technology being put into use as AT (and/or as a research tool) in the field of dementia care [34].

## The Research Base Needs to Keep Pace With Changes in AT Use

According to these 3 drivers of AT evolution, we now turn to methods the AT research community may wish to adopt to help AT keep pace with the demands of the future. Alongside the change in how AT is used has been a change in how AT is researched. A recent systematic review noted that very few of the published trials of AT to support older adults were of a gold standard for evidential trials (ie, randomized or clinical controlled trials) [5]. Further work has been done to create research protocols using the action-design-research methodology, which incorporates codesign into its structure, but these are yet to be tested in a controlled trial [35]. There is also a lack of consistency in the choice of outcome measures to allow comparison between trials [36].

One of the potential benefits of cocreation/codesign is to attempt to mitigate the phenomenon of a drop off in the use of new technology, which often occurs a few weeks after introduction in trials; it is suggested that the influence of codesign may work to embed AT into daily life in such a way that it inspires longevity of use [5].

## Learning from Cocreation: the Norfolk Experience

As mentioned, the county of Norfolk in the United Kingdom contains districts of exceptionally high old age dependency, such as North Norfolk [31], a large coastal county situated in the east of England with a population of approximately 1 million. With areas of both coastal and urban deprivation, combined with a high old age dependency, Norfolk is, in our opinion, an ideal location to conduct research into the evolution of health and social care service design. We recently conducted a patient and public involvement exercise across Norfolk on the topic of the transformation of health care with technology; the first theme of this exercise was based on the use of AT and remote monitoring [37].

Overall, the listening exercise showed that people are keen to engage in discussion around the use, design, and barriers to AT. The participants felt it was important to capture these views to inform future research and service design. Both AT users (ie, patients) and their carers recognized the benefits of using AT, with 2 key benefits highlighted being the reassurance provided to the individual around being safe at home and the reduction of stress on the National Health Service (NHS), which is particularly relevant at a time when it is recognized the NHS is under maximum strain [38]. The reassurance gained by AT provided in a virtual ward setting went further than safety, which was also based on the safety felt by knowing one is being monitored by a health care professional and could have easy access back to hospital if need be.

There is a wealth of evidence in the research literature that multiple subtypes of AT trialed among older adults with frailty show promising results, although it is acknowledged that these results have been at a small scale and are most impressive in cases of mild to moderate frailty [5,39].

This listening exercise also highlighted the key concern of the public around falling victim to scams. In this regard, a codesign process could be important to explore how to reassure the public that this risk can be mitigated, given that older adults are particularly vulnerable to scams that rely on the impersonation of authority figures [40].

## Cocreation Examples in AT Research

Encouragingly, the existing research base does seem to already integrate cocreation (ie, involving current or potential future service users) as a philosophy of trial design [5,35]. Cocreation is important to enable access to all. Our previous report and others have demonstrated the variation of digital literacy, internet access, and the impact of generational challenges (the youngest older adults self-describe as more “tech savvy” than the oldest older adults) [37,41]. There is also evidence that national programs can have a positive impact on digital literacy for older adults, as long as they do not treat all older adults as starting from the same knowledge base [42]. It is necessary to explore design considerations to address these potential inequalities of access.

There are many positive examples of the principle of cocreation being used in the field of AT, ranging from a novel robot design appropriate for many users to the customization of AT to provide bespoke solutions in a health care setting [43,44].

Public engagement would be a good way to explore the key motivating factors for technology users. One of our focus groups reported that one barrier to motivation was a reluctance to engage with new technology due to the fear of getting it wrong. However, the participants were also able to suggest a potential enabler to overcome this barrier: using peer-to-peer support. Indeed, this technique has been shown to help overcome similar barriers to older adults’ digital engagement in the health arena [37,45].

Other tools to enhance the use of AT have also been tested, including a novel collaborative tool that uses expert allied health professionals as a critical part of the codesign process [46]. The inherent aim of this approach is to maximize the dialog between providers and consumers of AT to increase the likelihood of a positive outcome.

Thus, there are multiple reasons to support the extensive use of cocreation for both research trials and for pragmatically enhancing the adoption of existing AT. We have already identified local services that are providing support around digital support/training on leaving the hospital (eg, digitally based shopping) that could potentially be expanded to help overcome this barrier.

## Summary

A quarter-century is a fair span of time by which to measure change, and the change in what constitutes AT has indeed been mighty over that period [3,5]. This change has occurred on many levels. At the simplest level, there are now more older adults living with disability who have a need of the latest 2024 version of 1999 AT equivalents, just as much as their forerunners would have back in the last millennium [4]. At the next quantum of change, there are not only more older adults but there is also a higher percentage of older adults who require AT due to those without a condition- or injury-specific disability nevertheless struggling to maintain independent living owing to frailty associated with age [6].

Advances in AT have been driven by opportunities greater than a priority need to broaden scope. These advances have been driven by the expansion of the envelope of what is possible, as new technologies that were the science fiction topics of our youth become the common-place technology of today.

To maximize the true potential of AT, we recommend that research trialists embrace the use of the principle of cocreation as a vehicle to enhance not only AT uptake but also the longevity of its impact [5,32].

## Limitations of This Viewpoint

While we have endeavored to back up our opinions with evidence from a variety of sources, we acknowledge that we have not challenged our central view that AT is an overall force for good in the battle to enable older adults to live independently. We note there is a counterview, with valid concerns that AT has potential downsides as well as benefits. These concerns include the potential for AT to undermine rather than enhance autonomy and the way in which AI tools trained on the shared characteristics of the majority population may struggle to handle diversity [47,48].

Although we have held up AT as a way to enable aging in place, we have not discussed potential public health strategies to ensure a lower health dependency in later life (potentially obviating the need for AT), an approach that may be most relevant to lower- and middle-income countries [49].

We have also not discussed alternatives to aging in place, such as assisted living facilities, multigenerational living arrangements, or long-term care facilities, but wish to acknowledge that these are equally valid preferred choices for some individuals [50,51].

It is also important to acknowledge that the effects of the aging process vary immensely among individuals and older adults are a diverse and heterogenous group, many of whom do not require any form of care.

However, we believe there has never been a time where there is a more pressing need for high-quality research into AT usage to address these concerns and to make aging in place a reality for the many worldwide rather than just the privileged few.

## Abbreviations

AI: artificial intelligence
AT: assistive technology
CCT: closed-caption technology
NHS: National Health Service
OADR: old age dependency ratio
SPA: State Pension age
